# Use of concomitant variceal embolization and prophylactic antiplatelet/anticoagulative in transjugular intrahepatic portosystemic shunting: A retrospective study of 182 cirrhotic portal hypertension patients

**DOI:** 10.1097/MD.0000000000008678

**Published:** 2017-12-08

**Authors:** Yingmei Tang, Sheng Zheng, Jinhui Yang, Weimin Bao, Lihong Yang, Yingchun Li, Ying Xu, Jing Yang, Yuyun Tong, Jinhang Gao, Chengwei Tang

**Affiliations:** a Department of Gastroenterology and Yunnan Research Center for Liver Diseases, The 2nd Affiliated Hospital of Kunming Medical University; b Department of Gastroenterology, Yunnan Provincial 3rd People's Hospital; c Department of General Surgery, Yunnan Provincial 1st People's Hospital; d Department of Interventional Radiology, The 2nd Affiliated Hospital of Kunming Medical University, Kunming, Yunnan; e Department of Gastroenterology, West China Hospital, Sichuan University, Chengdu, Sichuan, China.

**Keywords:** cirrhosis, fluency stenting, portal hypertension, prediction, transjugular intrahepatic portosystemic shunting, treatment outcome

## Abstract

Transjugular intrahepatic portosystemic shunting (TIPS) is an effective treatment modality for refractory variceal bleeding and ascites in patients with cirrhotic portal hypertension (CPH). Variceal rebleeding and shunt dysfunction are major post-TIPS morbidities. This study aimed to retrospectively evaluate the effectiveness and safety of use of concomitant variceal embolization and prophylactic antiplatelet/anticoagulative in patients with CPH undergoing TIPS. Between October 2006 and October 2011, 182 patients with CPH were retrospectively and consecutively hospitalized for elective TIPS with Fluency stenting. Concomitant variceal embolization was given after establishing the shunt. Subcutaneous heparin was given after TIPS and replaced by oral clopidogrel, aspirin, or warfarin for at least 6 months. Main outcome measures included shunt patency rate, recurrence of CPH (rebleeding and/or refractory ascites), hepatic encephalopathy (HE) frequency, and post-TIPS survival. The cumulative primary patency rate was 96%, 94%, 90%, 88%, and 88% at 6, 12, 24, 36, and 48 months, respectively. Shunt stenosis occurred in 16 (9%) patients, gastrointestinal (GI) rebleeding in 32 (17.5%) patients, recurrence of refractory ascites 44 (48%) patients, HE in 42 (23%) patients, and death in 36 (20%) patients during the follow-up period. Use of concomitant variceal embolization and prophylactic antiplatelet/anticoagulative was associated with a favorable shunt patency and a low risk of GI rebleeding.

## 1 Introduction

Liver cirrhosis (LC) is a common public health concern worldwide and occurs frequently in the East Asian population due to a high prevalence of chronic hepatitis B and C infection.^[1]^ LC results in 300,000 deaths per year as the sixth leading cause of death in China.^[2]^ In addition to liver dysfunction, cirrhotic portal hypertension (CPH) is the major complication of LC, which mainly contributes to esophagogastric varices and bleeding (EGVB) and refractory ascites.^[2]^ As a serious complication, EGVB is usually controlled by medical and/or endoscopic treatment, but these 2 modalities are associated with a high risk of recurrent bleeding.^[3]^ Ascites refractory to medical intervention may also cause more serious complications and require a more aggressive intervention.

Since performance of the first successful endovascular procedure in 1988,^[4]^ transjugular intrahepatic portosystemic shunting (TIPS) has been widely applied for treating CPH refractory to medical and/or endoscopic treatment, and this approach is usually regarded as a bridging treatment to liver transplantation due to the high risk of post-TIPS hepatic encephalopathy (HE).^[5]^ In addition to worsened gut-derived hyperammonemia, shunt stenosis, and stricture usually occur in approximately 50% of patients 0.5 to 1 year after TIPS and lead to recurrence of variceal bleeding and refractory ascites.^[6]^ Use of covered stent, such as Fluency with polytetrafluoroethylene, is reported to improve the patency rate with a favorable safety profile.^[7,8]^

Similar to but with a greater hemostatic effect as compared to ligation and sclerotherapy, concomitant variceal embolization is an effective treatment modality to control acute EGVB in patients with CPH.^[9]^ Previous studies suggested that TIPS combined with variceal embolization significantly reduce the risk of variceal bleeding as compared to TIPS without embolization.^[10]^ Shunt failure in vascular intervention practice, especially in early-onset cases, mainly results from in-stent thrombosis; use of prophylactic antiplatelet/ anticoagulative may help improve the stent patency but be subject to a high risk of bleeding. In percutaneous coronary intervention (PCI), use of polymer-free, drug-coated stent followed by a short-course antiplatelet therapy showed a favorable efficacy and safety profile.^[11]^

The primary objective of this study was to retrospectively evaluate the effectiveness and safety of use of concomitant variceal embolization and prophylactic antiplatelet/anticoagulative in patients with CPH undergoing TIPS with a covered stent.

## 2 Materials and methods

### 2.1 Patients

**Figure 1:**
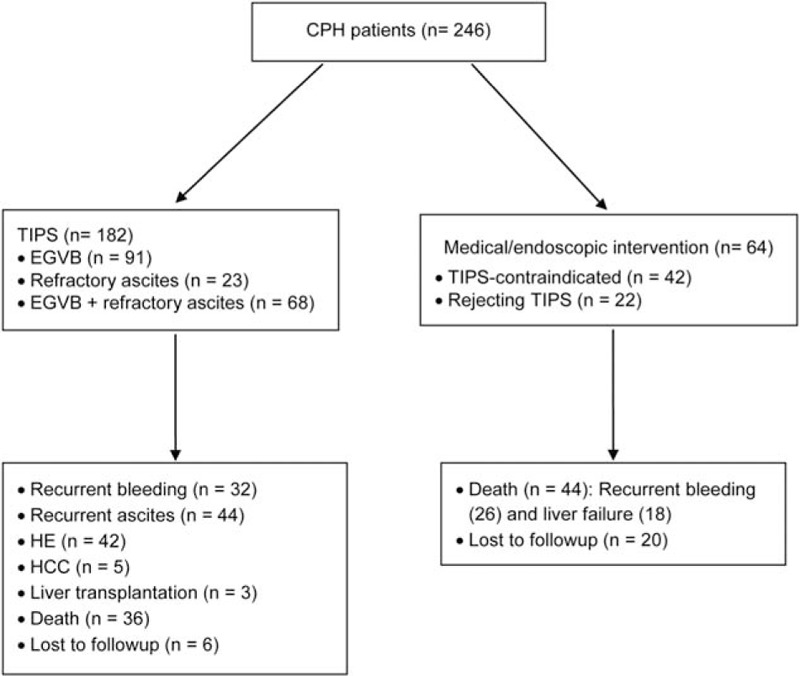
Treatment assignment of patients with CPH (n = 246). CPH = cirrhotic portal hypertension, EGVB = esophagogastric varices and bleeding, HE = hepatic encephalopathy, TIPS = transjugular intrahepatic portosystemic shunting.

Two hundred forty-six patients with CPH, who were consecutively hospitalized for elective or emergency TIPS at our Center for Liver Disease between October 2006 and October 2011, were included in this retrospective study (Fig. 1). The indications for TIPS were as follows: diagnosis of CPH; acute or recurrent EGVB, ascites or pleural effusion refractory to medical (unresponsive to high-dose diuretics and sodium restriction), and/or endoscopic intervention; a liver function reserve of Child-Pugh class B or C; and planned for liver transplantation. The contraindications were as follows: with complicating serious cardiopulmonary impairment; moderate or severe pulmonary hypertension (>35 mm Hg); serious organic renal insufficiency; refractory HE; multiple liver cysts; portal thrombosis or tumor thrombosis on the planned puncture access; portal cavernoma; a tumor located in proximity to the proposed puncture track; serious biliary obstruction; uncontrollable sepsis; or serious coagulopathy. Supportive treatment included restriction of dietary protein intake, rehydration, transfusion, and albumin supplementation. All patients or their legal representatives voluntarily gave written informed consent before undergoing TIPS.

### 2.2 Transjugular intrahepatic portosystemic shunting and concomitant variceal embolization

The anatomy of the liver and the portal and hepatic veins was examined using ultrasonography (Philips Medical Systems, Bothell, WA), 256-detector computed tomography (Brilliance iCT; Philips Medical Systems, Best, The Netherlands), and 1.5-T ultraconductive magnetic resonance imaging (Siemens Healthcare, Munich, Germany). The patient was positioned supine with the head turned toward the left side, with the heart rate, blood pressure, and saturation of peripheral oxygen continuously monitored. Under local anesthesia with 2% lidocaine, the right internal jugular vein was punctured at the midpoint on the lateral margin of the sternocleidomastoid muscle or 2 to 2.5 cm below the mandibular angle.

The puncture needle (Cook Medical, Bloomington, IN) was inserted at an angle of 30° to 45° and a depth of 3 to 5 cm. A super-slippery guide wire was advanced into the inferior vena cava, and the puncture access was dilated using a 10 F sheath dilator (Cook Medical). A Rosch-Uchida transjugular liver access set (Cook Medical) was selectively introduced into the right or left hepatic vein, and the puncture needle tip was adjusted to establish a shunt in connection with the portal vein (PV). Alternatively, the puncture needle was advanced into the retrohepatic inferior vena cava in proximity to the secondary hilum to insert the shunt into the PV if the hepatic vein was disorientated or obstructed by the fibrotic tissue shown on digital subtraction angiography (Siemens Healthcare) with 300 mg/mL iohexol (Yangtze River Pharmaceutical Group, Suzhou, China). Contrast radiography was repeated to confirm shunt patency, variceal embolization, positioning of the stent, requirement of an additional bare stent, and portal inflow. A 0.035i super-slippery guide wire (Terumo Corporation, Tokyo, Japan) was advanced through the main trunk of the PV to guide a 5 F Cobra angiographic catheter (Terumo Corporation) into the splenic or superior mesenteric vein for patients who had undergone splenectomy.

The varices of the gastric coronary vein, short veins, and esophageal veins were embolized using 3 to 12-mm coils (Cook Medical) and a gelatine sponge released through a 0.035i super-rigid exchange guide wire (Bard Medical, Covington, GA). The portohepatic shunt was dilated using a 6 to 8-mm balloon dilator (Bard Medical), and the 8 to 9 F Fluency self-expandable, endovascular, ultrathin expanded PTFE-covered NiTi alloy stent with a 2-mm bare part on both ends was deployed in the shunt. The Fluency stents were 6 to 10 mm in diameter (n = 172; 6 mm, n = 2; 7 mm, n = 13; 8 mm, n = 130; 10 mm, n = 27), at a length of 60 to 80 mm after expansion; the stent size was mainly selected based on the patient's body mass index and hepatic venous pressure gradient and were in length. Metallic bare stents at a length of 40 to 80 mm (n = 19; 6 mm, n = 2; 7 mm, n = 3; 8 mm, n = 8; 10 mm, n = 6) were also placed in the case of a long shunt. The PV pressure was recorded in mm Hg before and after shunting.

### 2.3 Antiplatelet/anticoagulative and follow-up

Subcutaneous injection of 4250-U low-molecular-weight heparin sodium was given every 12 h for 7 consecutive days and replaced by oral medications with clopidogrel sulfate [n = 95 (52.2%); 75 mg for patients with a platelet count above 100 × 10^9^/L, 50 mg for 50–100 × 10^9^/L, and 25 mg for 30–50 × 10^9^/L], enteric-coated aspirin [n = 64 (35.2%); daily dose of 100 mg for above 100 × 10^9^/L, 50 mg for 50–100 × 10^9^/L and 25 mg for 30–50 × 10^9^/L] as prophylactic antiplatelet therapy. For patients with pre-existing or treatment-emergent PV thrombosis, warfarin once daily [n = 9 (4.9%); down- or uptitrated from 3 mg (0.75, 1.5, 2.5, 3, and 4.5 mg) was prescribed to maintain the international normalized ratio (INR) at a range of 2–3] for at least 6 months. In patients with a platelet count below 30 × 10^9^/L and/or an INR above 2 (n = 14 [7.7%]), no anticoagulative or antiplatelet therapy was given; antiplatelet/anticoagulative medication would discontinued if uncontrolled gastrointestinal rebleeding occurred.

Patients were closely followed up at outpatient clinics 1, 3, 6, 12, 24, 36, and 48 months after TIPS using routine hematologic, clinical biochemistry, and coagulation function tests as well as upper gastrointestinal endoscopy, Doppler ultrasonography, computed tomography, magnetic resonance imaging, and digital subtraction angiography.

### 2.4 Main outcome measures and definitions

Main outcome measures included shunt patency rate, portal hypertension recurrence, HE frequency, and post-TIPS survival. The presence of shunt stenosis was suspected if the maximum shunt blood flow rate was <50 cm/s or the shunt diameter was <50%; the PV blood flow rate was <20 cm/s; the portosystemic gradient (PSG) was >11.8 mm Hg^[12]^; or portal hypertension recurred.^[13]^ Shunt revision was done, if shunt stenosis was confirmed on contrast angiography, using balloon dilation and/or second-look stenting. Recurrence of portal hypertension was defined as post-TIPS re-emergence of EGVB and/or refractory ascites. HE was staged according to the West-Haven criteria.^[14]^ Post-TIPS survival analyses included progression to primary hepatocellular carcinoma, receipt of liver transplantation, death, and loss to follow-up (failing to attend 2 consecutive scheduled visits).

### 2.5 Statistical analysis

The SPSS 17.0 statistical software package (SPSS Inc, Chicago, IL) was used for statistical analysis. All continuous data were expressed as mean ± standard deviation, and the means were compared using the paired Student *t* test. All categorical data were expressed as count (percentage) and compared using the Fisher exact probability test. Cumulative shunt patency rate, HE frequency, and survival data were analyzed using the Kaplan–Meier method. A 2-sided *P* value <.05 was considered statistically significant.

## 3 Results

### 3.1 Clinical and operative data

Out of 246 patients with CPH, 182 patients underwent TIPS with Fluency stenting. The other 64 patients, who were unsuitable for or rejected TIPS, received medical and/or endoscopic intervention; 44 patients died of recurrent bleeding (n = 26) or liver failure (n = 18); and the other 20 patients were lost to follow-up. Clinical and operative data for patients with CPH undergoing TIPS (n = 182) are shown in Table 1. Emergency TIPS was performed in 20 (11%) patients due to a first-time massive EGVB. TIPS with Fluency stenting was successfully completed in a single attempt in all patients, involving the right PV branch in 158 patients and the left PV branch in 24 patients.

**Table 1:**
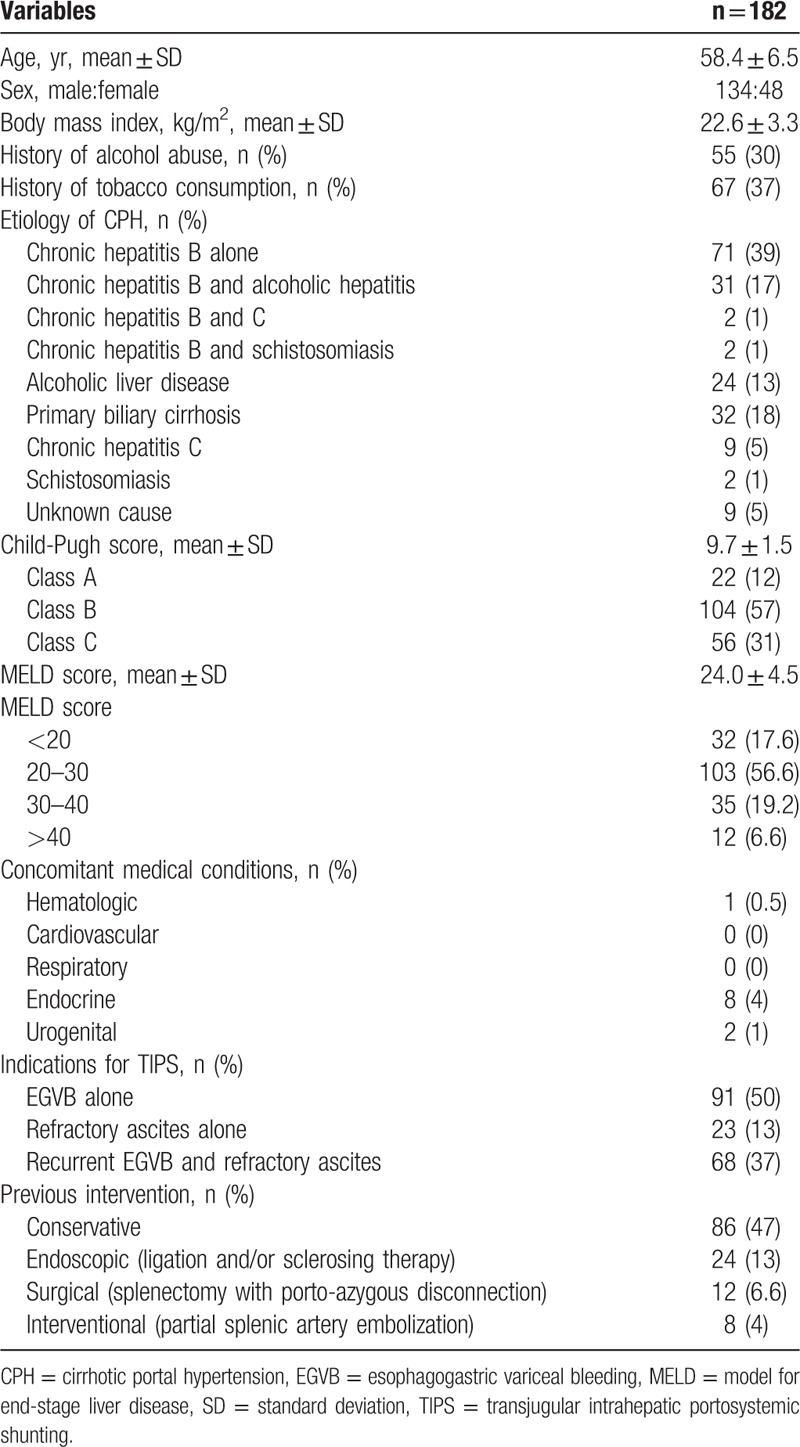
Clinical and operative data of patients with cirrhotic portal hypertension (n = 182).

### 3.2 Post-transjugular intrahepatic portosystemic shunting hemodynamic changes

Hemodynamic results are shown in Table 2. PV pressure and PSG significantly decreased immediately after TIPS (both *P* < .001). The caliber of the PV and the splenic vein (SP) also decreased significantly, accompanied by a significant increase in PV main trunk blood flow velocity (all *P* < .001).

**Table 2:**
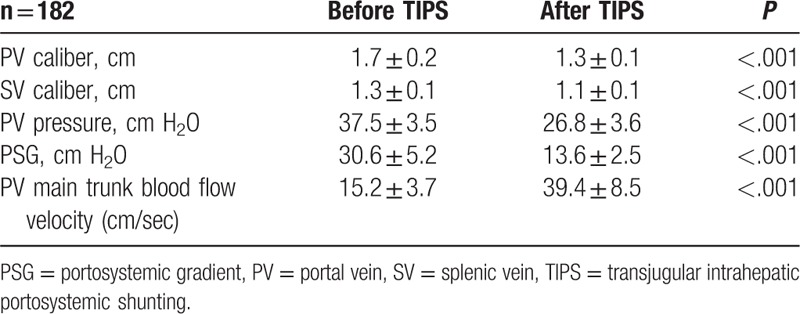
Post-transjugular intrahepatic portosystemic shunting hemodynamic changes (mean ± standard deviation).

### 3.3 Shunt patency

**Figure 2:**
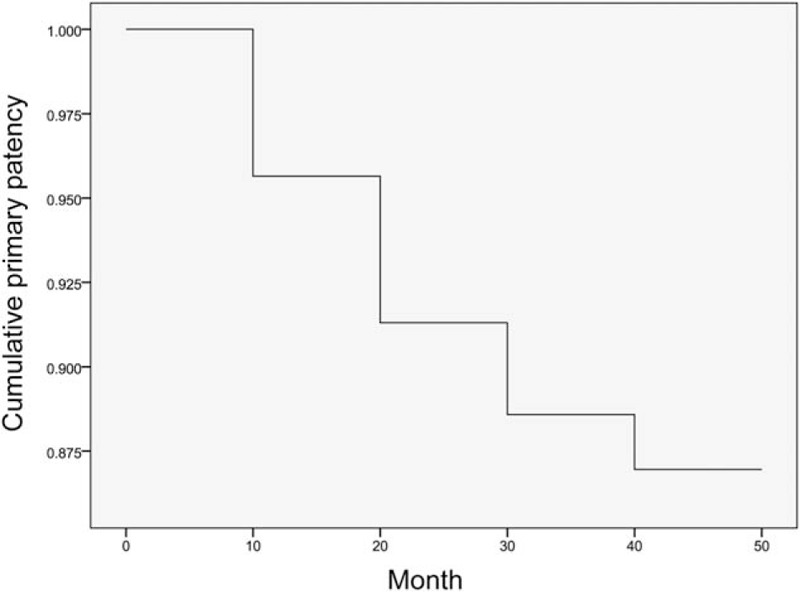
The Kaplan-Meier curve for cumulative primary patency rate.

The cumulative primary patency rate was 96%, 94%, 90%, 88%, and 88% at 6, 12, 24, 36, and 48 months, respectively (Fig. 2). During the follow-up period, 17 (9%) patients experienced shunt stenosis and obstruction at a mean time of 13.2 ± 8.0 months after TIPS and as early as postoperative day 10 due to shunt thrombosis. Chief manifestations were recurrence of pre-existing CPH-associated symptoms, including melena (n = 7), hematemesis (n = 4), and ascites (n = 6). Repeated PV contrast angiography identified in-stent thrombosis (n = 7) and stent capping by the venous wall (n = 10). Shunt stenosis resolved after balloon dilation in 14 patients, whereas additional stenting was performed in 1 patient and second-look stenting in the other PV branch was performed in 1 patient due to a sharp stent angulation. The overall primary patency rate was 91%, and the secondary patency rate was 99%. The risk of shunt stenosis was not significantly correlated with the baseline Child-Pugh classification (class A vs B vs C, 18% vs 16% vs 20%, *P* > .05). Clopidogrel was given to 95 (52%) patients, aspirin to 64 (35%) patients, and warfarin 9 (5%) patients; no anticoagulative or antiplatelet therapy was given in 14 (8%) patients. As compared to no medication, use of antiplatelet/anticoagulative therapy significantly decreased the risk of stent stenosis [10% (17/168) vs 36% (5/14), *P* = .005]. Moreover, the frequency of shunt stenosis was similar among patients having received clopidogrel, aspirin, or warfarin [12% (11/95) vs 9% (6/64) vs 0% (0/9), *P* = .529].

### 3.4 Portal hypertension recurrence

Data for recurrence of gastrointestinal bleeding (32/182, 17.5%) are shown in Table 3. Seven patients had early recurrence (<3 months after TIPS) without shunt stenosis, in whom rebleeding was successfully controlled with medication (discontinuation of anticoagulative and antiplatelet therapy and use of proton pump inhibitor). Twenty-five patients had late recurrence (≥3 months after TIPS), 15 out of whom had variceal rebleeding; 10 patients with EGVB underwent second-look stenting, whereas 5 patients rejected further treatment, resulting in 3 cases of deaths and 2 cases of recurrent bleeding.

**Table 3:**
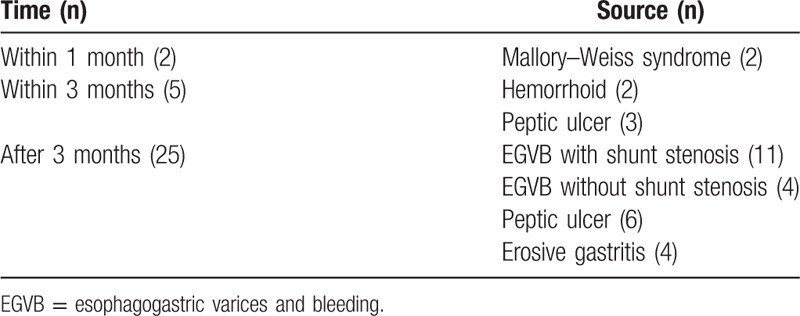
Time and source of gastrointestinal rebleeding (n = 32).

Pre-existing refractory ascites (n = 23) dissolved within 1 week after TIPS in 7 (30%) patients, resolved within 1 to 2 weeks in 15 (65%) patients, and remained unchanged in 1 (4%) patient. Among 68 patients who had variceal bleeding with complicating mild/moderate ascites, ascites dissolved in 15 (22%) patients within 2 weeks, resolved in 46 (71%) patients, and remained unchanged in 7 (10%) patients. Refractory ascites recurred in 48% (44/91) of patients due to liver function decompensation or shunt dysfunction.

### 3.5 Post-transjugular intrahepatic portosystemic shunting hepatic encephalopathy and survival data

**Figure 3:**
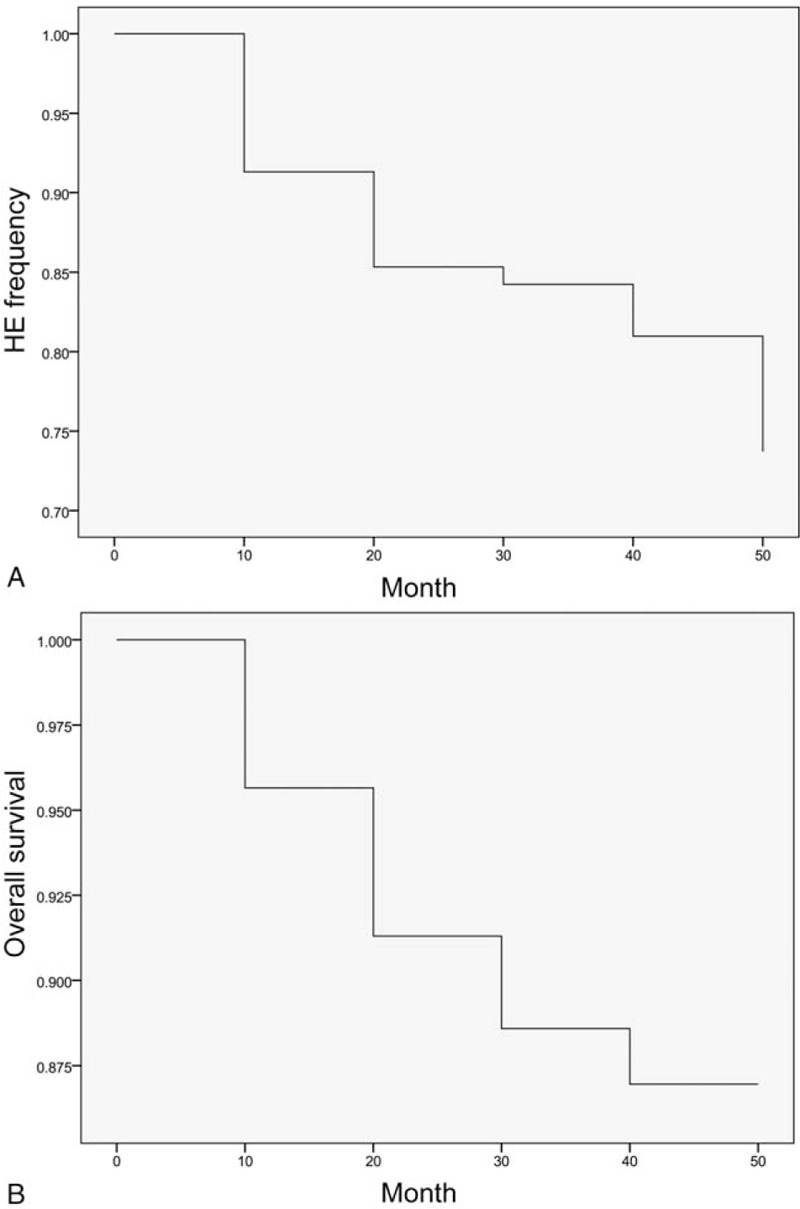
The Kaplan-Meier curves for (A) HE frequency and (B) overall survival. HE = hepatic encephalopathy.

Overall HE occurred in 42 (23%) patients after TIPS (Table 4; Fig. 3A). Stage I/II HE (n = 23) significantly resolved after restriction of dietary protein intake and medication with oral lactulose; stage III/IV HE (n = 19) resolved in 7 patients after supportive treatment but progressed to liver failure and death in 12 patients without shunt stenosis. Use of a 10-mm vs 8-mm stent (10 mm vs 8 mm, 30% vs 23%) and a shunt access to the right vs left PV branch (24% vs 17%) were associated with a significantly higher risk of HE (both *P* < .01). Patients with a baseline model for end-stage liver disease score of 30 to 40 or ≥40 had a significantly higher risk of HE than those with scores of <20 and 20 to 30 (<20 vs 20−30 vs 30−40 vs ≥40, 19% vs 21% vs 29% vs 42%, *P* < .01). In addition, 50 (28%) patients reached the survival analysis endpoints within a median follow-up period of 12.5 months (range, 3–60) months) (Table 4; Fig. 3B).

**Table 4:**
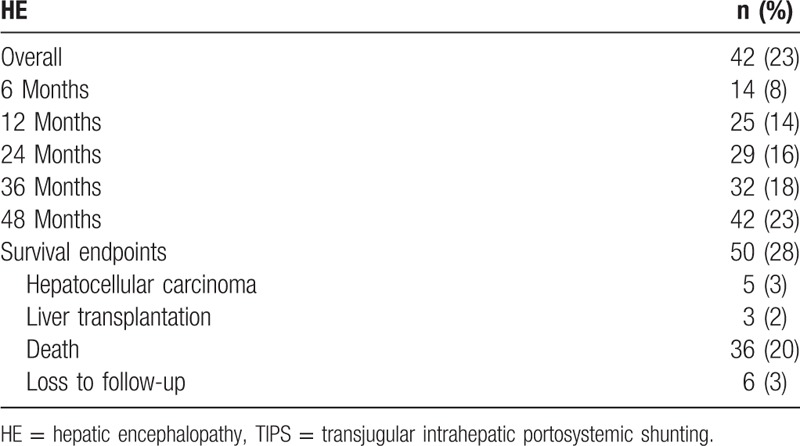
Post-transjugular intrahepatic portosystemic shunting hepatic encephalopathy and survival data.

## 4 Discussion

As the major limitation of TIPS, shunt dysfunction mainly results from in-stent stenosis and manifests as variceal rebleeding and re-emergence of refractory ascites. Use of a covered stent graft is known to improve the shunt patency and decrease the risk of TIPS dysfunction [2-year, 44%; risk ratio 0.60, 95% confidence interval (CI, 0.38,0.96)], without increasing the risk of HE [0.89 (0.53–1.49)] or mortality (70% vs 67.5%), as compared to that of a bare stent (63.6%).^[6]^ Concomitant embolization of portosystemic collateral vein, such as the coronary vein, also helps prevent shunt dysfunction (96.2% vs 82.0%, *P* = .019) and variceal rebleeding within the first 6 months after TIPS (96.2% vs 82.0%, *P* = .019).^[10,15]^ Moreover, antiplatelet and/or anticoagulative therapy has a controversial role in prevention of TIPS dysfunction although this prophylactic regimen has a well-documented efficacy and safety profile in the practice of PCI.^[11,16]^ To the best of our knowledge, this study was the first report regarding evaluation of concomitant variceal embolization and antiplatelet/anticoagulative therapy in unselected patients with CPH undergoing TIPS.

A PSG <11.8 mm Hg is associated with a significantly lower risk of recurrent variceal bleeding.^[12]^ In addition, TIPS is associated with a short-term (<3 months) increase in cardiac output and venous backflow in proportion to shunt caliber, which may aggravate underlying pulmonary hypertension and/or congestive heart failure.^[17]^ In our patients, PV pressure, PSG, and PV and SP caliber decreased significantly after TIPS with 8- or 10-mm Fluency stenting, with favorable therapeutic effects on controlling refractory EGVB and bleeding and minimal cardiopulmonary adverse events.

Shunt patency depends on multiple factors, including patient characteristics, stent size, covered graft, and operator's expertise.^[18]^ Wu et al^[19]^ reported in 114 patients with CPH that Fluency stenting resulted in a mean portal venous pressure reduction from 2.499 ± 0.588 to 1.764 ± 0.294 cm Hg, with patency rates of 86.7% and 75.2% at 1 and 2 years, respectively. A single-center randomized trial further showed that the cumulative stenosis rates with a 8-mm covered Fluency stent were 6.9%, 11.5%, 19.1%, 26.0%, and 35.9% at 1, 2, 3, 4, and 5 years, with significantly higher restenosis rates in patients with bare stents at these time points (27.6%, 37.0%, 49.6%, 59.8%, and 74.8%, respectively).^[20]^ In contrast, the cumulative stenosis frequency of stent at a variable size was relatively lower in our cohort (6%, 10%, 12%, and 12% at 1, 2, 3, and years, respectively). Moreover, our results showed that pre-existing liver function impairment was not predictive of the long-term shunt stenosis.

Combination of variceal embolization can control rebleeding from collateral vessels in patients with CPH. Chen et al^[10]^ reported that TIPS with coronary vein embolization (n = 54) was associated with a higher primary shunt patency rate than TIPS without embolization (n = 52) at 6 months (96.2% vs 82.0%, *P* = .019) but a lower risk of recurrent bleeding (5.7% vs 20.0%, *P* = .029), although this advantage was not observed afterwards. However, a meta-analysis showed that TIPS with variceal embolization could not reduce shunt dysfunction [odds ratio (OR) 1.26, 95% CI 0.76–2.08, *P* = .38], encephalopathy (OR 0.81, 95% CI 0.46–1.43, *P* = .47), and death (OR 0.90, 95% CI 0.55–1.47, *P* = .68), but could significantly decrease the risk of variceal rebleeding (OR 2.02, 95% CI 1.29–3.17, *P* = .002).^[21]^ Well-designed randomized, controlled trials with adequate statistical power are needed to warrant this finding especially when a covered stent is used.

Use of a covered stent can reduce shunt dysfunction; however, polymer coverage mainly inhibits in-stent pseudointimal hyperplasia at a late stage rather than hepatic or PV thrombosis at an early stage.^[22,23]^ In-stent thrombosis is a clinically significant event possibly causing shunt dysfunction and requiring a second-look intervention, although it occurred at a low frequency (4%) in our cohort. In the practice of PCI, long-term dual antiplatelet therapy with or without anticoagulation is evidenced to decrease the risk of stent failure.^[24]^ Our preliminary results showed that use of antiplatelet/anticoagulative therapy significantly decreased the risk of shunt stenosis, regardless of the prophylactic regimen, as compared to no medication. It should be borne in mind that the patients having received no medication were not comparable to those having received as the former had a lower platelet count and/or a prolonged INR. However, this finding along with previous literature regarding percutaneous coronary stenting encourages validation of the effectiveness of additional antiplatelet/ anticoagulative therapy in patients with CPH undergoing TIPS.

Variceal rebleeding and/or refractory ascites can recur due to shunt dysfunction and/or liver disease progression and in a close association with the endpoint events, including death and liver transplantation. Frequency of variceal bleeding significantly varied among previous reports. Risk factors include patient characteristics (especially underlying liver condition), stent-graft coverage and size, time of performing TIPS, and technical expertise. Sauerbruch et al^[25]^ inserted 8-mm covered stent into 90 patients with CPH, the great majority of whom had a liver function reserve of child class A or B, with 7% experiencing rebleeding within 2 years, as compared to 26% in patients receiving medical treatment alone (*P* = .002). García-Pagán et al^[26]^ reported a 1-year actuarial probability of rebleeding at 3% in decompensated patients with CPH after early use of TIPS with a covered stent as compared to 50% in patients on pharmacotherapy with banding (*P* < .001). In a recently published multicenter randomized trial, a 0% proportion of patients in the TIPS group had variceal rebleeding within a median follow-up of 23 months.^[27]^ In our cohort, the proportion of patients experiencing gastrointestinal rebleeding was relatively higher (17.5%); however, variceal rebleeding with or without shunt stenosis accounted approximately 50% of rebleeding patients and mainly occurred 3 months after TIPS. Early and nonvariceal rebleeding in our cohort might be associated with prophylactic use of antiplatelet/ anticoagulative therapy as evidenced by resolution after medication withdrawal. Similar to the practice of PCI, the benefit and risk of prophylactic antiplatelet/anticoagulative therapy needs to be balanced in further controlled clinical trials.

TIPS is superior to paracentesis in controlling ascites (recurrence rate, 42% vs 89%) and delaying the need for liver transplantation.^[28]^ TIPS with covered stenting can significantly improve the survival of patients with refractory ascites as compared to that with bare stenting.^[29]^ Our results showed that TIPS with Fluency stenting was effective at controlling refractory ascites and ascites with complicating EGVB. A relatively greater proportion (48%) of patients had post-TIPS recurrence of refractory ascites than those in previous reports (Narahara et al,^[30]^ 13.3%; Riggio et al,^[31]^ 8/10-mm, 60%/7%; Henderson et al,^[32]^ 33%). Recurrence of refractory ascites shares risk factors with post-TIPS rebleeding. The variation in frequency of refractory ascites recurrence could be attributed to heterogeneity in patient characteristics and stenting technique.

HE is the most serious complication secondary to TIPS with an incidence rate of 20% to 30% mainly due to gut-derived neurotoxin bypass and progressive deterioration of pre-existing liver insufficiency.^[33]^ TIPS is reported to increase the risk of early (within 1 year) HE occurrence rather than that in the long term as compared to medical intervention alone.^[27]^ Our results showed that mild HE usually occurs as transient controllable events within 3 months of TIPS, whereas moderate or severe HE normally emerges as refractory morbidities 6 to 12 months after TIPS with a poor prognosis mainly due to liver disease progression. A larger shunt size (stent caliber) was shown to be associated with a significantly higher risk of HE after TIPS, consistent with previous reports.^[34,35]^ The PV left branch is normally used as TIPS access for its lower procedural risk, better patency, and reduced post-TIPS HE.^[36,37]^ Underlying liver function reserve and disease progression are also documented to be closely associated with the risk of post-TIPS HE occurrence and survival.^[38]^ Current clinical trial results demonstrated an insignificant benefit of TIPS over medical or endoscopic therapy for patients with decompensated CPH regardless of stent coverage or size,^[25,27,30]^ although the former technique showed a validated clinical benefit in controlling variceal bleeding/rebleeding and refractory ascites.

There were some limitations in this study. Firstly, as a retrospective study, the design of this study failed comparison between TIPS with concomitant variceal embolization and that without. Secondly, our results could not make a statically validated conclusion on use of prophylactic antiplatelet/anticoagulative therapy as a small proportion of patients with CPH received no prophylaxis due to the protocol-defined contraindications. Lastly, our patient cohort was highly heterogeneous in baseline characteristics, including pre-existing liver function reserve, concomitant coagulopathy, emergency versus elective TIPS, and shunt success versus failure, which might favor or disfavor TIPS efficacy and safety. However, this study reported the real-life outcomes of TIPS with concomitant variceal embolization and antiplatelet/anticoagulative prophylaxis in unselected decompensated patients with CPH.

In conclusion, additional variceal embolization in TIPS helped reduce the risk of variceal rebleeding in decompensated patients with CPH with bleeding from collateral vessels. Use of prophylactic antiplatelet/anticoagulative therapy, if cautiously given in patients with a platelet count above 30 × 10^9^/L and/or an INR <2, showed a benefit in decreasing shunt stenosis, the major cause of shunt dysfunction which results in recurrent variceal bleeding and refractory ascites. Early medically controllable gastrointestinal bleeding was a major safety concern regarding antiplatelet/anticoagulative prophylaxis. Efficacy and safety of concomitant variceal embolization and antiplatelet/anticoagulative prophylaxis are further to be validated in randomized, controlled studies regarding unselected decompensated patients with CPH undergoing TIPS with a covered stent.

## References

[1] Huo T Wu JC Hwang SJ Factors predictive of liver cirrhosis in patients with chronic hepatitis B: a multivariate analysis in a longitudinal study. Eur J Gastroenterol Hepatol 2000;12:687–93. 10912490 10.1097/00042737-200012060-00019

[2] Wang SB Wang JH Chen J Natural history of liver cirrhosis in south China based on a large cohort study in one center: a follow-up study for up to 5 years in 920 patients. Chin Med J (Engl) 2012;125:2157–62. 22884146

[3] Primignani M Carpinelli L Preatoni P Natural history of portal hypertensive gastropathy in patients with liver cirrhosis. The New Italian Endoscopic Club for the study and treatment of esophageal varices (NIEC). Gastroenterology 2000;119:181–7. 10889167 10.1053/gast.2000.8555

[4] Rossle M Richter GM Noldge G New non-operative treatment for variceal haemorrhage. Lancet 1989;2:153. 10.1016/s0140-6736(89)90201-8 2567908

[5] Qi XS Bai M Yang ZP Selection of a TIPS stent for management of portal hypertension in liver cirrhosis: an evidence-based review. World J Gastroenterol 2014;20:6470–80. 24914368 10.3748/wjg.v20.i21.6470 PMC4047332

[6] Perarnau JM Le Gouge A Nicolas C Covered vs. uncovered stents for transjugular intrahepatic portosystemic shunt: a randomized controlled trial. J Hepatol 2014;60:962–8. 24480619 10.1016/j.jhep.2014.01.015

[7] Yang Z Han G Wu Q Patency and clinical outcomes of transjugular intrahepatic portosystemic shunt with polytetrafluoroethylene-covered stents versus bare stents: a meta-analysis. J Gastroenterol Hepatol 2010;25:1718–25. 21039832 10.1111/j.1440-1746.2010.06400.x

[8] Jirkovsky V Fejfar T Safka V Influence of the secondary deployment of expanded polytetrafluoroethylene-covered stent grafts on maintenance of transjugular intrahepatic portosystemic shunt patency. J Vasc Interv Radiol 2011;22:55–60. 21106389 10.1016/j.jvir.2010.09.016

[9] Bian S Tian XG Hu JH Percutaneous transhepatic variceal embolization combined with endoscopic ligation for the prevention of variceal rebleeding. J Dig Dis 2013;14:388–95. 23432941 10.1111/1751-2980.12049

[10] Chen S Li X Wei B Recurrent variceal bleeding and shunt patency: prospective randomized controlled trial of transjugular intrahepatic portosystemic shunt alone or combined with coronary vein embolization. Radiology 2013;268:900–6. 23657891 10.1148/radiol.13120800

[11] Urban P Meredith IT Abizaid A Polymer-free drug-coated coronary stents in patients at high bleeding risk. N Engl J Med 2015;373:2038–47. 26466021 10.1056/NEJMoa1503943

[12] Montomoli J Holland-Fischer P Bianchi G Body composition changes after transjugular intrahepatic portosystemic shunt in patients with cirrhosis. World J Gastroenterol 2010;16:348–53. 20082481 10.3748/wjg.v16.i3.348 PMC2807956

[13] Ricci P Cantisani V Lombardi V Is color-Doppler US a reliable method in the follow-up of transjugular intrahepatic portosystemic shunt (TIPS)? J Ultrasound 2007;10:22–7. 23396711 10.1016/j.jus.2007.02.005 PMC3478715

[14] Koziarska D Wunsch E Milkiewicz M Mini-Mental State Examination in patients with hepatic encephalopathy and liver cirrhosis: a prospective, quantified electroencephalography study. BMC Gastroenterol 2013;13:107. 23815160 10.1186/1471-230X-13-107 PMC3716589

[15] Tesdal IK Filser T Weiss C Transjugular intrahepatic portosystemic shunts: adjunctive embolotherapy of gastroesophageal collateral vessels in the prevention of variceal rebleeding. Radiology 2005;236:360–7. 15955858 10.1148/radiol.2361040530

[16] Theilmann L Sauer P Roeren T Acetylsalicylic acid in the prevention of early stenosis and occlusion of transjugular intrahepatic portal-systemic stent shunts: a controlled study. Hepatology 1994;20:592–7. 8076917

[17] Pudil R Praus R Hulek P Transjugular intrahepatic portosystemic shunt is associated with significant changes in mitral inflow parameters. Ann Hepatol 2013;12:464–70. 23619264

[18] Patidar KR Sydnor M Sanyal AJ Transjugular intrahepatic portosystemic shunt. Clin Liver Dis 2014;18:853–76. 25438287 10.1016/j.cld.2014.07.006 PMC4251783

[19] Wu Q Jiang J He Y Transjugular intrahepatic portosystemic shunt using the FLUENCY expanded polytetrafluoroethylene-covered stent. Exp Ther Med 2013;5:263–6. 23251280 10.3892/etm.2012.776 PMC3523954

[20] Wang L Xiao Z Yue Z Efficacy of covered and bare stent in TIPS for cirrhotic portal hypertension: a single-center randomized trial. Sci Rep 2016;6:21011. 26876503 10.1038/srep21011 PMC4753460

[21] Qi X Liu L Bai M Transjugular intrahepatic portosystemic shunt in combination with or without variceal embolization for the prevention of variceal rebleeding: a meta-analysis. J Gastroenterol Hepatol 2014;29:688–96. 24117967 10.1111/jgh.12391

[22] Heinzow HS Lenz P Kohler M Clinical outcome and predictors of survival after TIPS insertion in patients with liver cirrhosis. World J Gastroenterol 2012;18:5211–8. 23066315 10.3748/wjg.v18.i37.5211 PMC3468853

[23] Cura M Cura A Suri R Causes of TIPS dysfunction. AJR Am J Roentgenol 2008;191:1751–7. 19020247 10.2214/AJR.07.3534

[24] Yeh RW Secemsky EA Kereiakes DJ Development and validation of a prediction rule for benefit and harm of dual antiplatelet therapy beyond 1 year after percutaneous coronary intervention. JAMA 2016;315:1735–49. 27022822 10.1001/jama.2016.3775 PMC5408574

[25] Sauerbruch T Mengel M Dollinger M Prevention of rebleeding from esophageal varices in patients with cirrhosis receiving small-diameter stents versus hemodynamically controlled medical therapy. Gastroenterology 2015;149:660.e1–8.e1. 25989386 10.1053/j.gastro.2015.05.011

[26] Garcia-Pagan JC Caca K Bureau C Early use of TIPS in patients with cirrhosis and variceal bleeding. N Engl J Med 2010;362:2370–9. 20573925 10.1056/NEJMoa0910102

[27] Holster IL Tjwa ET Moelker A Covered transjugular intrahepatic portosystemic shunt versus endoscopic therapy + β-blocker for prevention of variceal rebleeding. Hepatology 2016;63:581–9. 26517576 10.1002/hep.28318

[28] Salerno F Camma C Enea M Transjugular intrahepatic portosystemic shunt for refractory ascites: a meta-analysis of individual patient data. Gastroenterology 2007;133:825–34. 17678653 10.1053/j.gastro.2007.06.020

[29] Clark W Golkar F Luberice K Uncovering the truth about covered stents: is there a difference between covered versus uncovered stents with transjugular intrahepatic portosystemic shunts? Am J Surg 2011;202:561–4. 21944293 10.1016/j.amjsurg.2011.06.021

[30] Narahara Y Kanazawa H Fukuda T Transjugular intrahepatic portosystemic shunt versus paracentesis plus albumin in patients with refractory ascites who have good hepatic and renal function: a prospective randomized trial. J Gastroenterol 2011;46:78–85. 20632194 10.1007/s00535-010-0282-9

[31] Riggio O Ridola L Angeloni S Clinical efficacy of transjugular intrahepatic portosystemic shunt created with covered stents with different diameters: results of a randomized controlled trial. J Hepatol 2010;53:267–72. 20537753 10.1016/j.jhep.2010.02.033

[32] Henderson JM Boyer TD Kutner MH Distal splenorenal shunt versus transjugular intrahepatic portal systematic shunt for variceal bleeding: a randomized trial. Gastroenterology 2006;130:1643–51. 16697728 10.1053/j.gastro.2006.02.008

[33] Berlioux P Robic MA Poirson H Pre-transjugular intrahepatic portosystemic shunts (TIPS) prediction of post-TIPS overt hepatic encephalopathy: the critical flicker frequency is more accurate than psychometric tests. Hepatology 2014;59:622–9. 24620380 10.1002/hep.26684

[34] Fanelli F Angeloni S Salvatori FM Transjugular intrahepatic portosystemic shunt with expanded-polytetrafuoroethylene-covered stents in non-cirrhotic patients with portal cavernoma. Dig Liver Dis 2011;43:78–84. 20637712 10.1016/j.dld.2010.06.001

[35] Rossle M Gerbes AL TIPS for the treatment of refractory ascites, hepatorenal syndrome and hepatic hydrothorax: a critical update. Gut 2010;59:988–1000. 20581246 10.1136/gut.2009.193227

[36] Chen L Xiao T Chen W Outcomes of transjugular intrahepatic portosystemic shunt through the left branch vs. the right branch of the portal vein in advanced cirrhosis: a randomized trial. Liver Int 2009;29:1101–9. 19386025 10.1111/j.1478-3231.2009.02016.x

[37] Bai M He CY Qi XS Shunting branch of portal vein and stent position predict survival after transjugular intrahepatic portosystemic shunt. World J Gastroenterol 2014;20:774–85. 24574750 10.3748/wjg.v20.i3.774 PMC3921486

